# From Association to Prediction: Translational Barriers in Pain Biomarker Research

**DOI:** 10.3390/jpm16070374

**Published:** 2026-07-13

**Authors:** Gustavo Fabregat-Cid, Natalia Escrivá-Matoses, José De Andrés

**Affiliations:** 1 Multidisciplinary Pain Management Department, University General Hospital, 46014 Valencia, Spain; 2 Surgery Department, Medical School, University of Valencia, 46010 Valencia, Spain; jose.andres@uv.es; 3 Primary Care Department, Conselleria de Sanitat, 46010 Valencia, Spain; escriva_nat@gva.es; 4 Multidisciplinary Pain Clinic, Vithas Virgen del Consuelo Hospital, 46007 Valencia, Spain

**Keywords:** pain biomarkers, chronic pain, predictive biomarkers, precision pain medicine, biomarker validation, translational research, neuroimaging, machine learning, personalized medicine, spinal cord stimulation

## Abstract

Pain biomarkers have been proposed as potential tools to improve patient stratification, treatment selection, and individualized therapeutic strategies in chronic pain. However, despite an increasing volume of research across neuroimaging, electrophysiological, and molecular domains, their translation into clinical practice remains limited. A central challenge lies in the conceptual and methodological misalignment between biomarker discovery and clinical applicability. Many studies labelled as “predictive” rely on measurements obtained during or after intervention, small sample sizes, or lack of external validation, limiting their ability to inform real-world decision-making. In addition, the distinction between predictive, monitoring, and mechanistic biomarkers is often blurred, further complicating interpretation and implementation. This Perspective examines why many candidate pain biomarkers, although biologically informative, have not yet become clinically actionable tools for treatment selection. We distinguish between associative, mechanistic, monitoring, preventive, and truly predictive biomarkers using clinically relevant examples from pain research, and we outline the methodological requirements needed to translate biomarker discovery into precision pain medicine. We argue that the field requires a more rigorous framework for defining and validating predictive biomarkers, encompassing appropriate timing of measurement, robust study design, external validation, and patient-relevant endpoints. Without this framework, pain biomarker research risks continuing to generate biologically informative but clinically non-actionable findings that do not advance individualized therapeutic decision-making.

## 1. Introduction

Chronic pain is a highly prevalent and heterogeneous condition, affecting multiple biological, psychological, and social domains [1,2]. Conservative estimates suggest that chronic pain affects approximately one in five adults globally, although prevalence varies substantially according to case definition, population, and geographical context [3,4]. Despite advances in therapeutic options, treatment outcomes remain highly variable, and the ability to predict individual response to specific interventions is limited. Clinical decision-making is still largely based on symptom patterns and trial-and-error approaches, which often result in suboptimal outcomes and prolonged patient suffering [5].

In this context, biomarkers, broadly defined as measurable indicators of biological processes, pathological processes, or responses to an intervention, have been proposed as tools to improve patient stratification, guide treatment selection, and enable more personalized approaches to pain management [6,7]. In chronic pain specifically, candidate biomarkers have been investigated across multiple domains, including neuroimaging, electrophysiology, autonomic physiology, quantitative sensory testing, and molecular profiling, with the aim of characterizing pain mechanisms, stratifying patients, and predicting treatment response [8,9,10].

Advances in neuroimaging and systems neuroscience have provided insights into the central mechanisms of pain, identifying large-scale brain networks involved in sensory, affective, and cognitive processing [11,12,13]. Similarly, molecular and immune markers have been explored as indicators of neuroinflammatory and systemic processes associated with chronic pain states [14]. Together, these developments have strengthened interest in the use of biomarkers to support more mechanism-informed and personalized approaches to pain management.

The current state of pain biomarker research is therefore heterogeneous rather than uniformly negative. Candidate biomarkers span multiple domains, including neuroimaging, quantitative sensory testing, electrophysiology, autonomic physiology, inflammatory and immune markers, metabolomic and proteomic profiles, genetic and epigenetic variation, and psychosocial or behavioral variables [8,9,10,15]. Recent work has emphasized that single-modality biomarkers are unlikely to capture the multidimensional nature of chronic pain, and that composite or multimodal signatures may be more informative than isolated biological measures [9,10]. In this regard, large-scale evidence has shown that biological markers derived from blood immunoassays, brain and bone imaging, and genetics can predict medical conditions associated with chronic pain with moderate accuracy, although they perform less well for self-reported pain when used alone [16]. Importantly, predictive performance improves substantially when biological markers are integrated with psychosocial factors, reinforcing the need for biopsychosocial and multimodal biomarker models rather than purely biological signatures [16]. Several biomarker domains have generated recurrent findings in pain research, although their clinical utility remains uneven. Neuroimaging studies have repeatedly implicated distributed networks involved in sensory-discriminative, affective, salience, cognitive-control, and default-mode processing, sometimes described as components of the “pain matrix” or broader pain-related brain networks [11,12,13,17,18]. However, these patterns are not specific to pain and have shown stronger performance in controlled experimental pain than in heterogeneous clinical pain states. Similarly, immune and inflammatory studies have reported alterations in cytokines and neuroimmune mediators, including IL-6, TNF-α, IL-1β, chemokines, and related inflammatory pathways, across nociceptive, neuropathic, postsurgical, and chronic widespread pain conditions [8,14,15]. These findings support a mechanistic role for neuroimmune activation and sensitization, but their specificity and individual-level predictive value remain limited. Quantitative sensory testing, endogenous pain modulation, electrophysiology, and omics-based approaches have also provided important mechanistic and phenotypic information, although most remain better suited for stratification or mechanistic characterization than for treatment-guiding prediction [8,10,15,16,19,20,21]. Representative biomarker domains, main reported findings, potential clinical uses, and key translational barriers are summarized in Table 1.

Despite these advances, the translation of pain biomarkers into clinical practice remains limited. Few biomarkers have demonstrated sufficient validity, reproducibility, and clinical utility to support decision-making in routine care [8,21]. As a result, treatment selection in chronic pain continues to rely largely on clinical judgment rather than objective biological indicators.

A central issue lies in the misalignment between biomarker discovery and clinical applicability. Many studies report associations between biological signals and pain states or outcomes, yet these findings are often interpreted as predictive without meeting the methodological requirements necessary for true prediction [22,23]. In particular, the timing of measurement, lack of external validation, and limited sample sizes constrain the ability of these biomarkers to inform real-world clinical decisions.

Furthermore, the distinction between predictive, monitoring, and mechanistic biomarkers is frequently unclear, contributing to conceptual ambiguity and inflated perceptions of clinical readiness in pain biomarker research [7].

These challenges are particularly evident in the field of neuromodulation, where spinal cord stimulation (SCS) has emerged as an established therapy for chronic pain but continues to show substantial inter-individual variability in treatment response [24,25]. This variability has driven growing interest in identifying biomarkers capable of predicting therapeutic response. A growing body of literature has explored electrophysiological, neuroimaging, and molecular markers in this context [26,27,28,29]. However, many of these studies rely on signals obtained during or after stimulation, small exploratory cohorts, or models lacking external validation, limiting their ability to inform pre-treatment clinical decision-making [8]. As a result, SCS represents a paradigmatic example of the broader challenges in pain biomarker research: despite promising signals, few candidate biomarkers meet the criteria required for clinically actionable prediction.

In this context, the aim of this article is not to provide a comprehensive systematic review of the field, but rather to offer a critical perspective on the current landscape of pain biomarker research. Specifically, we seek to examine the methodological and conceptual limitations that hinder clinical translation and to clarify the requirements that biomarkers must meet to be considered truly predictive and clinically actionable.

We argue that advancing toward precision pain medicine requires a shift from associative and exploratory findings to rigorously validated, clinically relevant biomarkers. This transition depends on clear conceptual definitions, appropriate timing of measurement, robust study design, and the use of meaningful clinical endpoints capable of supporting real-world decision-making.

## 2. Methodological Barriers to Predictive Biomarkers

The limited clinical translation of pain biomarkers is largely driven by a set of recurring methodological and conceptual challenges that constrain their predictive utility. These limitations are not specific to individual biomarker domains but reflect broader issues in study design, interpretation, and classification, which are consistently observed across neuroimaging, electrophysiological, and molecular research in chronic pain [12].

A first critical barrier concerns the timing of biomarker measurement. For a biomarker to be clinically predictive, it must be obtained prior to treatment initiation and provide information that can guide decision-making. However, a substantial proportion of studies rely on intraoperative or post-intervention measurements, which may reflect biological response rather than baseline susceptibility [8]. This distinction is particularly relevant in interventional pain therapies, where physiological signals recorded during stimulation are often interpreted as predictors of outcome despite being inherently dependent on treatment exposure. Such signals may provide valuable mechanistic insights into treatment response. However, because they are obtained during or after intervention, they cannot inform pre-treatment stratification and therefore do not constitute predictive biomarkers in a clinically actionable sense.

A second major limitation relates to study design, particularly sample size, model stability, and validation. Many studies in pain biomarker research are based on small and highly selected cohorts, limiting statistical power and increasing the risk of overfitting, especially when multivariable or machine learning approaches are applied [30,31]. In this context, performance metrics such as accuracy or area under the curve (AUC) may appear promising but are often unstable and sensitive to sample composition. The absence of independent external validation further limits the generalizability of these findings, making it difficult to translate reported predictive performance into real-world clinical settings. Established reporting and methodological frameworks, such as the TRIPOD statement, emphasize the need for transparent reporting and adequate validation of prediction models [32].

A third barrier is the conceptual misclassification of biomarkers. Although the distinction between predictive, monitoring, and mechanistic biomarkers is well established [7], these categories are frequently conflated in pain research. Biomarkers reflecting treatment-induced changes or disease-related biological processes are often described as predictive, despite lacking the capacity to guide treatment selection. This issue is compounded by the use of heterogeneous endpoints and inconsistent definitions of response, which further obscure the intended role of each biomarker type. More broadly, the overinterpretation of exploratory findings in small or heterogeneous datasets has been widely recognized as a limitation in biomedical research, contributing to challenges in reproducibility and translation [33].

These methodological concerns are reflected in the broader pain biomarker literature, where many proposed markers remain associative rather than truly predictive. Neuroimaging “pain signatures,” for example, have demonstrated sensitivity to experimentally evoked pain but have not consistently translated into robust, generalizable clinical predictors [13]. A critical review has highlighted limitations in specificity and clinical applicability, emphasizing the risk of overinterpreting such markers as objective measures of pain [17]. Similarly, the widely cited Neurologic Pain Signature (NPS) demonstrated strong discrimination between painful and non-painful stimuli in controlled experimental settings, but its ability to predict clinical pain or treatment response remains uncertain [18].

At a population level, large-scale studies further illustrate these limitations. Biological markers alone often show modest predictive performance for chronic pain conditions, with substantial improvement only when psychosocial variables are incorporated, suggesting limited independent clinical utility [16]. In parallel, high-dimensional proteomic studies have identified numerous associations with chronic pain, yet predictive performance remains moderate and largely confined to observational risk models rather than treatment-guiding frameworks [20].

Even when stronger predictive signals are reported, they are frequently derived from controlled experimental models or highly individualized approaches, limiting generalizability to heterogeneous clinical populations [34,35]. Similarly, in related conditions such as osteoarthritis, multi-biomarker models typically demonstrate only modest discriminative performance despite extensive biomarker evaluation [36].

Together, these findings reinforce the notion that, despite substantial scientific advances, most pain biomarkers remain closer to associative indicators than to clinically actionable predictive tools.

## 3. Clinical Consequences

These methodological limitations have direct implications for clinical practice. Despite extensive research activity, few biomarkers are currently used to guide treatment selection in chronic pain [8,17,19], and clinical decision-making continues to rely largely on empirical approaches and clinician judgment [37].

This gap between research and practice limits the implementation of precision pain medicine, as patients are often selected for treatments without reliable biological indicators of response. Consequently, variability in treatment outcomes remains high across both pharmacological and interventional therapies, including neuromodulation, where response heterogeneity remains a major clinical challenge [24].

In addition, the overinterpretation of preliminary or methodologically limited findings may contribute to an inflated perception of clinical readiness [33,38,39]. Biomarkers derived from small or unvalidated studies are sometimes implicitly translated into clinical expectations, despite lacking reproducibility or generalizability [40]. This not only delays the identification and validation of robust biomarkers but also risks misdirecting research efforts and resource allocation.

As a result, the field faces a paradox in which increasing scientific output has not translated into proportionate clinical impact. Bridging this gap requires not only the identification of novel biomarkers, but also a more rigorous framework to evaluate their clinical relevance and applicability.

## 4. The Way Forward

Preventive strategies and the development of more effective therapies remain major unmet needs in pain medicine. However, these priorities should not be viewed as competing with biomarker research. Well-validated biomarkers may contribute to prevention by identifying individuals at higher risk of pain chronification, refining patient stratification in clinical trials, clarifying mechanisms relevant to therapeutic development, and enabling earlier or more targeted intervention. In this sense, predictive biomarkers should be understood not as a substitute for preventive or therapeutic innovation, but as tools that may help make such strategies more precise, testable, and clinically implementable [8,9,16,41].

### 4.1. Redefining Predictive Biomarkers

A central step toward improving translational progress in pain biomarker research is the establishment of clearer and more rigorous definitions of what constitutes a truly predictive biomarker. In many studies, biomarkers associated with pain severity, neurophysiological alterations, or treatment-related changes are implicitly interpreted as predictive despite lacking the capacity to inform therapeutic decision-making before intervention [17,18,42]. This distinction is critical, as predictive biomarkers are intended to identify individuals who are more likely to benefit from a specific treatment, rather than simply characterize disease state or biological response [7].

In precision medicine research, predictive biomarkers are typically defined by their ability to demonstrate a treatment–biomarker interaction capable of influencing therapeutic selection [43,44]. By contrast, much of the pain biomarker literature remains focused on association-based findings, often without formal evaluation of predictive performance or clinical utility [8,42]. As a result, biomarkers are frequently proposed as clinically relevant despite limited evidence that they improve treatment allocation or patient outcomes.

In pain biomarker research, these categories can be illustrated by different clinical scenarios. Associative biomarkers identify biological differences linked to pain presence, severity, or phenotype, such as altered brain connectivity, sensory profiles, or inflammatory mediator levels in patients with chronic pain [8,10,15]. Mechanistic biomarkers provide insight into biological pathways involved in nociception, sensitization, neuroimmune activation, or central plasticity, but do not necessarily guide clinical decisions [12,13,14]. Monitoring or pharmacodynamic biomarkers reflect treatment engagement or biological response after therapy has begun, such as stimulation-evoked neurophysiological signals during neuromodulation or longitudinal changes in inflammatory or sensory measures [27,29,42]. Preventive biomarkers aim to identify patients at increased risk of pain chronification, for example after surgery, trauma, or nerve injury [41]. Truly predictive biomarkers, in contrast, should help determine before a relevant therapeutic decision whether an individual patient is more likely to benefit from one treatment strategy than another [43,45,46]. Distinguishing these categories is essential because each requires different validation standards and supports different types of clinical decisions [7].

A more rigorous framework for predictive biomarker development in pain medicine should therefore incorporate several key principles: for biomarkers intended to guide initial treatment selection, measurement should occur before treatment initiation; for repeated or sequential treatments, early-response or post-intervention biomarkers may inform subsequent therapeutic decisions; candidate markers should be validated in independent and clinically heterogeneous populations; and endpoints should be clinically meaningful and relevant to patient-centered outcomes [32,45]. Importantly, predictive performance should be assessed not only through statistical association but also through reproducibility, calibration, and potential impact on clinical decision-making.

An additional conceptual shift may be required to move the field beyond biomarker discovery toward biomarker actionability. In pain medicine, the central question should not be whether a biological signal differs between patients with and without pain, or between responders and non-responders, but whether that signal can be positioned within a concrete clinical decision pathway. A biomarker becomes clinically meaningful only when it provides information that is unavailable from standard clinical assessment and can plausibly change management before the clinical decision it is intended to inform [7,43]. This implies that candidate biomarkers should be evaluated not only by statistical association or discrimination, but also by their incremental value over routine clinical variables, their timing relative to the therapeutic decision, and their potential to influence treatment allocation. Decision-analytic approaches, such as decision curve analysis, may be useful in this context because they explicitly assess whether predictive information provides net clinical benefit across clinically relevant decision thresholds [46].

From this perspective, pain biomarkers may be viewed along a translational maturity continuum. At one end are mechanistic or state biomarkers that improve biological understanding but do not directly guide care. At an intermediate level are monitoring or pharmacodynamic biomarkers that reflect treatment engagement or biological response after therapy has begun. At the highest level are clinically actionable predictive biomarkers, which identify, before treatment initiation, whether a patient is more likely to benefit from one therapeutic strategy than from another. This distinction is particularly important in interventional pain medicine and neuromodulation, where signals obtained during or after stimulation may be valuable for optimizing therapy but should not be conflated with pretreatment predictors of response.

Accordingly, future biomarker studies in pain should be designed around explicit clinical use-cases: for example, whether a biomarker helps select candidates for neuromodulation, choose between pharmacological and interventional strategies, identify patients unlikely to benefit from invasive treatment, or personalize stimulation parameters after implantation. Framing biomarker research around such decision points may help distinguish biologically interesting observations from tools with genuine potential to support precision pain medicine.

Ultimately, redefining predictive biomarkers in this way may help distinguish exploratory biological observations from clinically actionable tools, thereby facilitating a more realistic and translationally meaningful pathway toward precision pain medicine.

### 4.2. Minimum Requirements for Clinical Translation

Current evidence suggests that the transition from exploratory biomarker research to clinically actionable tools will require substantially more rigorous translational standards than those currently applied in much of the pain literature [8,39]. Although methodological heterogeneity is expected in an evolving field, several minimum requirements appear necessary to support meaningful clinical implementation.

First, predictive biomarkers should be evaluated in prospective and clinically representative populations rather than highly selected or narrowly defined cohorts. Chronic pain syndromes are intrinsically heterogeneous, encompassing biological, psychological, and social dimensions that may substantially influence treatment response [16,47]. Biomarkers developed in restricted experimental settings may therefore fail to generalize to routine clinical populations.

Second, external validation should be considered essential rather than optional. Prediction models developed within a single cohort frequently demonstrate reduced performance when applied to independent datasets, reflecting both overfitting and population-specific effects [22,31]. Despite this, external validation remains uncommon in pain biomarker studies, limiting confidence in reported predictive performance. Accordingly, biomarkers should be regarded as candidate biomarkers until they have demonstrated generalizability across independent populations and clinically meaningful applicability within the decision context for which they are proposed [8,40].

Third, clinically meaningful endpoints are required. Many biomarker studies rely on surrogate neurophysiological or imaging outcomes without demonstrating a clear relationship with patient-centered measures such as pain relief, functional improvement, quality of life, or reduction in disability. From a translational perspective, statistical association alone is insufficient if biomarkers do not ultimately improve clinically relevant decision-making or therapeutic outcomes [5].

Finally, reproducibility and transparency in analytical methods are critical, particularly in studies involving machine learning or high-dimensional biological data. Standardized reporting frameworks and transparent methodological practices are necessary to reduce bias and improve comparability across studies [32].

Taken together, these principles suggest that the future success of pain biomarkers will depend less on the identification of increasingly complex biological signals and more on the methodological robustness with which these signals are validated and translated into clinically meaningful applications.

Without rigorous validation standards, the field risks continuing to generate biologically interesting but clinically non-actionable findings. These minimum requirements are summarized in Table 2. Although several of these requirements are shared with biomarker research in other fields, they are particularly relevant in pain medicine because pain outcomes are subjective, multidimensional, context-dependent, and strongly influenced by psychological, behavioral, and social factors. This makes external validation, incremental value over clinical assessment, feasibility, and patient-centered endpoints especially important for pain biomarker translation.

Together, these requirements emphasize that biomarker development should be judged not only by biological plausibility or statistical association, but by whether the candidate biomarker improves clinically relevant decisions in a feasible, generalizable, and patient-centered manner.

### 4.3. Beyond Single-Modality Biomarkers

Another important challenge in pain biomarker research is the reliance on isolated biological signals to explain or predict highly multidimensional clinical phenomena. Chronic pain is shaped by complex interactions among sensory, emotional, cognitive, autonomic, and social factors, making it unlikely that a single biomarker modality will provide sufficient predictive accuracy across heterogeneous patient populations [48,49].

At present, no single pain biomarker explains a large proportion of variance in clinically relevant outcomes across heterogeneous patient populations [16,49]. The situation resembles other complex biological traits, where multiple biological, psychological, and contextual factors each contribute modestly and variably across individuals. For this reason, candidate biomarkers should not be evaluated in isolation, but according to their incremental value when added to existing clinical prediction models [32,46]. Demonstrating that a biomarker is statistically associated with an outcome is insufficient; studies should show whether its inclusion improves model performance, calibration, clinical utility, or patient stratification beyond information already available from standard clinical assessment.

This limitation has led to growing interest in multimodal approaches that integrate neuroimaging, electrophysiological, molecular, autonomic, and psychosocial data within unified analytical frameworks. In other areas of precision medicine, combining heterogeneous data sources has improved classification and prognostic performance compared with single-domain models alone [50,51,52]. A similar strategy may be necessary in pain medicine, where biological signals often capture only one dimension of a broader clinical phenotype.

Importantly, psychosocial and behavioral factors should not be viewed as confounders to be excluded, but rather as integral components of pain-related predictive models. Large-scale population studies have shown that the predictive performance of biological markers frequently improves when psychosocial variables are incorporated, reinforcing the multidimensional nature of chronic pain [16].

However, increasing model complexity also introduces important methodological challenges, including dimensionality reduction, reproducibility, interpretability, and risk of overfitting. As multimodal biomarker models become more sophisticated, transparent validation strategies and clinically interpretable outputs will become increasingly important to ensure meaningful translation into practice.

Ultimately, the future of precision pain medicine will likely depend not on the identification of a single “ideal” biomarker, but on the integration of complementary biological and clinical signals capable of supporting individualized therapeutic decisions.

### 4.4. Toward Clinically Actionable Precision Pain Medicine

Ultimately, the primary goal of pain biomarker research should not be limited to improving biological characterization of chronic pain, but rather to supporting clinically meaningful decision-making. A biomarker becomes clinically valuable only when it contributes to treatment selection, improves outcome prediction, or meaningfully changes patient management beyond existing clinical assessment alone [44].

Importantly, the clinical value of biomarkers in pain medicine should not be restricted to predicting analgesic efficacy. Biomarkers may also be useful for predicting adverse effects, complications, tolerability, or treatment discontinuation [7,40]. In some contexts, safety-related outcomes such as nausea, sedation, infection, neurological adverse events, or poor tolerability may be more biologically tractable than the multidimensional experience of pain itself. Expanding biomarker research to include both benefit and harm may therefore provide a more realistic and clinically useful framework for personalized pain management [46].

The threshold for biomarker usefulness should also depend on the burden, risk, reversibility, and cost of the intervention being considered [40,46]. Biomarker-guided prediction may be most valuable when the therapeutic decision involves invasive procedures, high costs, delayed reversibility, substantial adverse-effect risk, or limited opportunities for empirical trial-and-error. By contrast, for low-risk, inexpensive, rapidly reversible treatments, the burden and cost of biomarker testing may outweigh its practical value unless it provides substantial incremental benefit over routine clinical assessment.

In this context, precision pain medicine should be understood not as the pursuit of increasingly complex biological signatures, but as the development of practical and reproducible tools capable of informing individualized therapeutic strategies. This distinction is particularly important in chronic pain, where treatment response is influenced by multidimensional factors that extend beyond isolated neurobiological mechanisms.

Accordingly, future translational efforts should prioritize clinically interpretable models, prospective validation in heterogeneous populations, and integration with real-world clinical workflows. Rather than focusing exclusively on biomarker discovery, the field may benefit from greater emphasis on implementation-oriented research aimed at determining whether biomarker-guided approaches improve patient outcomes, reduce therapeutic uncertainty, or optimize resource utilization.

Such a shift would help align biomarker research with the broader objectives of precision medicine, moving from descriptive biological associations toward actionable clinical tools capable of supporting personalized pain management.

## 5. Conclusions

Pain biomarker research has generated substantial scientific interest across neuroimaging, electrophysiological, molecular, and computational domains. However, despite significant advances in biomarker discovery, translation into clinically actionable tools remains limited. Many proposed biomarkers continue to rely on associative findings, small exploratory cohorts, or insufficient validation, reducing their capacity to support individualized therapeutic decision-making.

Advancing toward precision pain medicine will require a shift from descriptive biological associations to rigorously validated predictive frameworks grounded in methodological robustness, clinical relevance, and translational applicability. In this context, the future value of pain biomarkers will depend not only on technological innovation, but also on the ability to integrate biological and clinical information into reproducible tools capable of meaningfully improving patient care.

Rather than diminishing the relevance of pain biomarkers, this Perspective argues for a more precise and clinically grounded framework in which biomarker discovery, prevention, therapeutic development, and treatment selection are aligned toward patient-relevant outcomes.

The challenge is no longer simply discovering biomarkers but determining which biomarkers can meaningfully change clinical decisions and improve patient outcomes.

## Figures and Tables

**Table 1:** Representative pain biomarker domains, main reported findings, potential clinical uses, and key translational barriers.

Biomarker Domain	Representative Examples	Main Reported Findings in Pain Research	Potential Clinical Use	Main Translational Barriers
Neuroimaging biomarkers	Functional MRI signatures, resting-state connectivity, structural brain changes	Altered activity and connectivity have been reported in distributed pain-related networks, including somatosensory, insular, anterior cingulate, prefrontal, salience, default-mode, and sensorimotor networks. Experimental pain signatures can discriminate evoked pain in controlled settings, but generalization to heterogeneous clinical pain remains limited.	Pain phenotyping, mechanism-based stratification, prediction of treatment response	Limited specificity, variability in acquisition and preprocessing, small samples, uncertain generalizability to routine clinical pain
Electrophysiological biomarkers	EEG/MEG spectral features, evoked potentials, spinal or cortical signals, ECAP-related measures in neuromodulation	Chronic pain has been associated with altered oscillatory activity, cortical excitability, evoked responses, and functional connectivity. In neuromodulation, evoked spinal signals may reflect therapy engagement or stimulation effects, but are often measured during or after treatment exposure.	Objective monitoring, mechanistic characterization, therapy optimization	Often measured during or after intervention, limited pretreatment predictive value, lack of standardized thresholds
Quantitative sensory testing and endogenous modulation	Conditioned pain modulation, temporal summation, sensory thresholds	Impaired conditioned pain modulation, enhanced temporal summation, and altered sensory thresholds have been reported across several chronic pain conditions, supporting central sensitization or impaired endogenous inhibitory control in selected patient subgroups.	Phenotyping of pain mechanisms, stratification in neuropathic pain, treatment enrichment	Operator dependence, variability across protocols, limited implementation in routine care
Molecular and immune biomarkers	Cytokines, inflammatory mediators, metabolomic and proteomic profiles, neuroimmune markers	Altered levels of pro-inflammatory and anti-inflammatory mediators, including IL-6, TNF-α, IL-1β, chemokines, and neuroimmune pathways, have been described in nociceptive, neuropathic, postsurgical, and chronic widespread pain states. These findings support a role for immune activation and sensitization, but individual markers are heterogeneous and non-specific.	Identification of biological pathways, risk stratification, therapeutic target discovery	Low specificity, influence of comorbidities and systemic factors, limited replication and external validation
Genetic and epigenetic biomarkers	Candidate gene variants, genome-wide association signals, epigenetic signatures, microRNA profiles	Genetic and epigenetic studies have identified variants and regulatory mechanisms associated with pain susceptibility, chronification, and treatment response. However, most individual effects are small and likely require integration with clinical, psychosocial, and biological variables.	Susceptibility/risk assessment, mechanistic insight, potential treatment stratification	Small effect sizes, population heterogeneity, unclear individual-level clinical utility
Multimodal and computational biomarkers	Integrated models combining biological, imaging, electrophysiological, psychosocial, and clinical data	Multimodal approaches suggest that biological markers perform better when integrated with psychosocial, sensory, imaging, and clinical variables. Recent machine-learning and omics-based studies show promise, but many models remain exploratory and require external validation.	Personalized prediction, decision support, trial enrichment	Risk of overfitting, need for large datasets, interpretability, calibration, and prospective external validation
Neuroimaging biomarkers	Functional MRI signatures, resting-state connectivity, structural brain changes	Altered activity and connectivity have been reported in distributed pain-related networks, including somatosensory, insular, anterior cingulate, prefrontal, salience, default-mode, and sensorimotor networks. Experimental pain signatures can discriminate evoked pain in controlled settings, but generalization to heterogeneous clinical pain remains limited.	Pain phenotyping, mechanism-based stratification, prediction of treatment response	Limited specificity, variability in acquisition and preprocessing, small samples, uncertain generalizability to routine clinical pain
Electrophysiological biomarkers	EEG/MEG spectral features, evoked potentials, spinal or cortical signals, ECAP-related measures in neuromodulation	Chronic pain has been associated with altered oscillatory activity, cortical excitability, evoked responses, and functional connectivity. In neuromodulation, evoked spinal signals may reflect therapy engagement or stimulation effects, but are often measured during or after treatment exposure.	Objective monitoring, mechanistic characterization, therapy optimization	Often measured during or after intervention, limited pretreatment predictive value, lack of standardized thresholds
Quantitative sensory testing and endogenous modulation	Conditioned pain modulation, temporal summation, sensory thresholds	Impaired conditioned pain modulation, enhanced temporal summation, and altered sensory thresholds have been reported across several chronic pain conditions, supporting central sensitization or impaired endogenous inhibitory control in selected patient subgroups.	Phenotyping of pain mechanisms, stratification in neuropathic pain, treatment enrichment	Operator dependence, variability across protocols, limited implementation in routine care
Molecular and immune biomarkers	Cytokines, inflammatory mediators, metabolomic and proteomic profiles, neuroimmune markers	Altered levels of pro-inflammatory and anti-inflammatory mediators, including IL-6, TNF-α, IL-1β, chemokines, and neuroimmune pathways, have been described in nociceptive, neuropathic, postsurgical, and chronic widespread pain states. These findings support a role for immune activation and sensitization, but individual markers are heterogeneous and non-specific.	Identification of biological pathways, risk stratification, therapeutic target discovery	Low specificity, influence of comorbidities and systemic factors, limited replication and external validation
Genetic and epigenetic biomarkers	Candidate gene variants, genome-wide association signals, epigenetic signatures, microRNA profiles	Genetic and epigenetic studies have identified variants and regulatory mechanisms associated with pain susceptibility, chronification, and treatment response. However, most individual effects are small and likely require integration with clinical, psychosocial, and biological variables.	Susceptibility/risk assessment, mechanistic insight, potential treatment stratification	Small effect sizes, population heterogeneity, unclear individual-level clinical utility
Multimodal and computational biomarkers	Integrated models combining biological, imaging, electrophysiological, psychosocial, and clinical data	Multimodal approaches suggest that biological markers perform better when integrated with psychosocial, sensory, imaging, and clinical variables. Recent machine-learning and omics-based studies show promise, but many models remain exploratory and require external validation.	Personalized prediction, decision support, trial enrichment	Risk of overfitting, need for large datasets, interpretability, calibration, and prospective external validation

**Table 2:** Minimum requirements for clinically actionable pain biomarker studies.

Requirement	Rationale	Practical Implication
Clearly defined clinical use-case	A biomarker cannot be evaluated without knowing the decision it is intended to inform	Define whether the biomarker is intended for diagnosis, risk prediction, treatment selection, monitoring, safety prediction, or therapy optimization
Appropriate timing of measurement	Timing determines whether a biomarker can support initial treatment selection, monitoring, or sequential decision-making	Pretreatment markers are needed for initial treatment selection; post-intervention or early-response markers may inform repeated or sequential therapies
Incremental value over existing clinical models	A biomarker is useful only if it adds information beyond routine clinical assessment	Report whether the biomarker improves discrimination, calibration, net benefit, or clinical decision-making beyond standard clinical predictors
Clinically meaningful endpoint	Biomarkers should be linked to patient-relevant outcomes rather than surrogate changes alone	Use outcomes such as pain relief, function, quality of life, disability, adverse effects, complications, or treatment discontinuation
External validation and generalizability	Predictive performance within a single cohort may not translate to broader clinical populations	Validate in independent, clinically heterogeneous cohorts and report calibration and performance across relevant subgroups
Feasibility and proportionality	Biomarker testing should be justified by the risk, cost, burden, and reversibility of the clinical decision	Biomarkers may be more valuable before invasive, costly, or high-risk interventions than before low-risk empirical treatments
Candidate status until clinical validation	Biological plausibility or association alone does not establish clinical utility	Use the term “candidate biomarker” until generalizability, clinical applicability, and decision impact have been demonstrated

## Data Availability

No new data were created or analyzed in this study.

## References

[1] Turk D.C., Fillingim R.B., Ohrbach R., Patel K.V. (2016). Assessment of Psychosocial and Functional Impact of Chronic Pain. J. Pain.

[2] Treede R.D., Rief W., Barke A., Aziz Q., Bennett M.I., Benoliel R., Cohen M., Evers S., Finnerup N.B., First M.B. (2019). Chronic pain as a symptom or a disease: The IASP Classification of Chronic Pain for the International Classification of Diseases (ICD-11). Pain.

[3] Mills S.E.E., Nicolson K.P., Smith B.H. (2019). Chronic pain: A review of its epidemiology and associated factors in population-based studies. Br. J. Anaesth..

[4] Lurie J.M., Javaid A. (2024). Visualizing Global Chronic Pain. Anesth. Analg..

[5] Dworkin R.H., Turk D.C., Wyrwich K.W., Beaton D., Cleeland C.S., Farrar J.T., Haythornthwaite J.A., Jensen M.P., Kerns R.D., Ader D.N. (2008). Interpreting the clinical importance of treatment outcomes in chronic pain clinical trials: IMMPACT recommendations. J. Pain.

[6] Strimbu K., Tavel J.A. (2010). What are biomarkers?. Curr. Opin. HIV AIDS.

[7] Group F-NBW, Food and Drug Administration (US) (2016). BEST (Biomarkers, EndpointS, and other Tools) Resource.

[8] Davis K.D., Aghaeepour N., Ahn A.H., Angst M.S., Borsook D., Brenton A., Burczynski M.E., Crean C., Edwards R., Gaudilliere B. (2020). Discovery and validation of biomarkers to aid the development of safe and effective pain therapeutics: Challenges and opportunities. Nat. Rev. Neurol..

[9] Tracey I., Woolf C.J., Andrews N.A. (2019). Composite Pain Biomarker Signatures for Objective Assessment and Effective Treatment. Neuron.

[10] Eldabe S., Obara I., Panwar C., Caraway D. (2022). Biomarkers for Chronic Pain: Significance and Summary of Recent Advances. Pain Res. Manag..

[11] Baliki M.N., Apkarian A.V. (2015). Nociception, Pain, Negative Moods, and Behavior Selection. Neuron.

[12] Kuner R., Flor H. (2016). Structural plasticity and reorganisation in chronic pain. Nat. Rev. Neurosci..

[13] Zhang L.B., Chen Y.X., Li Z.J., Geng X.Y., Zhao X.Y., Zhang F.R., Bi Y.Z., Lu X.J., Hu L. (2024). Advances and challenges in neuroimaging-based pain biomarkers. Cell Rep. Med..

[14] Ji R.R., Nackley A., Huh Y., Terrando N., Maixner W. (2018). Neuroinflammation and Central Sensitization in Chronic and Widespread Pain. Anesthesiology.

[15] Mackey S., Aghaeepour N., Gaudilliere B., Kao M.C., Kaptan M., Lannon E., Pfyffer D., Weber K. (2025). Innovations in acute and chronic pain biomarkers: Enhancing diagnosis and personalized therapy. Reg. Anesth. Pain Med..

[16] Fillingim M., Tanguay-Sabourin C., Parisien M., Zare A., Guglietti G.V., Norman J., Petre B., Bortsov A., Ware M., Perez J. (2025). Biological markers and psychosocial factors predict chronic pain conditions. Nat. Hum. Behav..

[17] Mouraux A., Iannetti G.D. (2018). The search for pain biomarkers in the human brain. Brain.

[18] Wager T.D., Atlas L.Y., Lindquist M.A., Roy M., Woo C.W., Kross E. (2013). An fMRI-based neurologic signature of physical pain. N. Engl. J. Med..

[19] Baron R., Dickenson A.H., Calvo M., Dib-Hajj S.D., Bennett D.L. (2023). Maximizing treatment efficacy through patient stratification in neuropathic pain trials. Nat. Rev. Neurol..

[20] Li Z.Y., Ma Q., Zhang J., Yin R.Y., You J., Hao Q.Z., Wu X.R., Kang J.J., Wang L.B., Deng Y.T. (2025). Large-Scale Plasma Proteomics to Profile Pathways and Prognosis of Chronic Pain. Adv. Sci..

[21] Yarnitsky D., Granot M., Granovsky Y. (2014). Pain modulation profile and pain therapy: Between pro- and antinociception. Pain.

[22] Justice A.C., Covinsky K.E., Berlin J.A. (1999). Assessing the generalizability of prognostic information. Ann. Intern. Med..

[23] Riley R.D., Hayden J.A., Steyerberg E.W., Moons K.G., Abrams K., Kyzas P.A., Malats N., Briggs A., Schroter S., Altman D.G. (2013). Prognosis Research Strategy (PROGRESS) 2: Prognostic factor research. PLoS Med..

[24] Deer T.R., Mekhail N., Provenzano D., Pope J., Krames E., Leong M., Levy R.M., Abejon D., Buchser E., Burton A. (2014). The appropriate use of neurostimulation of the spinal cord and peripheral nervous system for the treatment of chronic pain and ischemic diseases: The Neuromodulation Appropriateness Consensus Committee. Neuromodulation.

[25] Kapural L., Yu C., Doust M.W., Gliner B.E., Vallejo R., Sitzman B.T., Amirdelfan K., Morgan D.M., Brown L.L., Yearwood T.L. (2015). Novel 10-kHz High-frequency Therapy (HF10 Therapy) Is Superior to Traditional Low-frequency Spinal Cord Stimulation for the Treatment of Chronic Back and Leg Pain: The SENZA-RCT Randomized Controlled Trial. Anesthesiology.

[26] De Andres J., Ten-Esteve A., Harutyunyan A., Romero-Garcia C.S., Fabregat-Cid G., Asensio-Samper J.M., Alberich-Bayarri A., Marti-Bonmati L. (2021). Predictive Clinical Decision Support System Using Machine Learning and Imaging Biomarkers in Patients With Neurostimulation Therapy: A Pilot Study. Pain Physician.

[27] Fabregat-Cid G., Cedeño D.L., Harutyunyan A., Rodríguez-López R., Monsalve-Dolz V., Mínguez-Martí A., Hernández-Cádiz M.J., Escrivá-Matoses N., Villanueva-Pérez V., Asensio Samper J.M. (2023). Effect of Conventional Spinal Cord Stimulation on Serum Protein Profile in Patients With Persistent Spinal Pain Syndrome: A Case-Control Study. Neuromodulation.

[28] Fabregat-Cid G., Cedeno D.L., De Andrés J., Harutyunyan A., Monsalve-Dolz V., Mínguez-Martí A., Escrivá-Matoses N., Asensio-Samper J.M., Carnaval T., Villoria J. (2024). Insights into the pathophysiology and response of persistent spinal pain syndrome type 2 to spinal cord stimulation: A human genome-wide association study. Reg. Anesth. Pain Med..

[29] Gopal J., Bao J., Harland T., Pilitsis J.G., Paniccioli S., Grey R., Briotte M., McCarthy K., Telkes I. (2025). Machine learning predicts spinal cord stimulation surgery outcomes and reveals novel neural markers for chronic pain. Sci. Rep..

[30] Riley R.D., Snell K.I., Ensor J., Burke D.L., Harrell F.E., Moons K.G., Collins G.S. (2019). Minimum sample size for developing a multivariable prediction model: PART II-binary and time-to-event outcomes. Stat. Med..

[31] Steyerberg E.W., Eijkemans M.J., Harrell F.E., Habbema J.D. (2000). Prognostic modelling with logistic regression analysis: A comparison of selection and estimation methods in small data sets. Stat. Med..

[32] Collins G.S., Reitsma J.B., Altman D.G., Moons K.G. (2015). Transparent Reporting of a multivariable prediction model for Individual Prognosis or Diagnosis (TRIPOD): The TRIPOD statement. Ann. Intern. Med..

[33] Ioannidis J.P. (2005). Why most published research findings are false. PLoS Med..

[34] Chowdhury N.S., Bi C., Furman A.J., Chiang A.K.I., Skippen P., Si E., Millard S.K., Margerison S.M., Spies D., Keaser M.L. (2025). Predicting Individual Pain Sensitivity Using a Novel Cortical Biomarker Signature. JAMA Neurol..

[35] Lee J.J., Jo S., Cho S., Woo C.W. (2026). Personalized brain decoding of spontaneous pain in individuals with chronic pain. Nat. Neurosci..

[36] Kraus V.B., Collins J.E., Hargrove D., Losina E., Nevitt M., Katz J.N., Wang S.X., Sandell L.J., Hoffmann S.C., Hunter D.J. (2017). Predictive validity of biochemical biomarkers in knee osteoarthritis: Data from the FNIH OA Biomarkers Consortium. Ann. Rheum. Dis..

[37] Swarm R.A., Youngwerth J.M., Agne J.L., Anitescu M., Are M., Buga S., Butler T., Chwistek M., Cleary J., Copenhaver D. (2025). Adult Cancer Pain, Version 2.2025, NCCN Clinical Practice Guidelines in Oncology. J. Natl. Compr. Cancer Netw..

[38] Kempf E., de Beyer J.A., Cook J., Holmes J., Mohammed S., Nguyên T.L., Simera I., Trivella M., Altman D.G., Hopewell S. (2018). Overinterpretation and misreporting of prognostic factor studies in oncology: A systematic review. Br. J. Cancer.

[39] Smothers C.G., Gandhi S.A., Chang J.H., Tahir S.M., Kelley J., Wang E., Fareed K., Mack H., Dong C., Witort M. (2026). Systematic review and meta-analysis of stroke blood biomarker data highlights need for more translational research methods. Nat. Commun..

[40] Turakhia M.P., Sabatine M.S. (2017). How We Evaluate Biomarker Studies. JAMA Cardiol..

[41] Sluka K.A., Wager T.D., Sutherland S.P., Labosky P.A., Balach T., Bayman E.O., Berardi G., Brummett C.M., Burns J., Buvanendran A. (2023). Predicting chronic postsurgical pain: Current evidence and a novel program to develop predictive biomarker signatures. Pain.

[42] Zebhauser P.T., Hohn V.D., Ploner M. (2023). Resting-state electroencephalography and magnetoencephalography as biomarkers of chronic pain: A systematic review. Pain.

[43] Simon R. (2010). Clinical trial designs for evaluating the medical utility of prognostic and predictive biomarkers in oncology. Pers. Med..

[44] Seymour L., Bogaerts J., Perrone A., Ford R., Schwartz L.H., Mandrekar S., Lin N.U., Litière S., Dancey J., Chen A. (2017). iRECIST: Guidelines for response criteria for use in trials testing immunotherapeutics. Lancet Oncol..

[45] Pepe M.S., Etzioni R., Feng Z., Potter J.D., Thompson M.L., Thornquist M., Winget M., Yasui Y. (2001). Phases of biomarker development for early detection of cancer. J. Natl. Cancer Inst..

[46] Vickers A.J., Elkin E.B. (2006). Decision curve analysis: A novel method for evaluating prediction models. Med. Decis. Mak..

[47] Turk D.C., Okifuji A. (2002). Psychological factors in chronic pain: Evolution and revolution. J. Consult. Clin. Psychol..

[48] Apkarian A.V., Baliki M.N., Geha P.Y. (2009). Towards a theory of chronic pain. Prog. Neurobiol..

[49] Fillingim R.B. (2017). Individual differences in pain: Understanding the mosaic that makes pain personal. Pain.

[50] Karczewski K.J., Snyder M.P. (2018). Integrative omics for health and disease. Nat. Rev. Genet..

[51] Osipov A., Nikolic O., Gertych A., Parker S., Hendifar A., Singh P., Filippova D., Dagliyan G., Ferrone C.R., Zheng L. (2024). The Molecular Twin artificial-intelligence platform integrates multi-omic data to predict outcomes for pancreatic adenocarcinoma patients. Nat. Cancer.

[52] Li C.X., Wheelock C.E., Sköld C.M., Wheelock Å.M. (2018). Integration of multi-omics datasets enables molecular classification of COPD. Eur. Respir. J..

